# Longitudinal Assessment of Ketamine-Assisted Community of Practice Therapy: A Retrospective 5-Year Study on Depression, Anxiety and PTSD

**DOI:** 10.1192/j.eurpsy.2026.11732

**Published:** 2026-07-31

**Authors:** A. Kuzma-Hunt, Z. Hamadeh, V. Tsang

**Affiliations:** 1 Department of Pathology and Laboratory Medicine; 2 Faculty of Medicine, University of British Columbia, Vancouver, Canada

## Abstract

**Introduction:**

Ketamine-assisted therapy shows rapid benefit in treatment-resistant depression, anxiety, and PTSD (Walsh et al. BJPsych Open 2022;8:e19). The Roots to Thrive Ketamine assisted Therapy (RTT-KaT) program is a unique 12-week program that combines 12 Community of Practice (group therapy) sessions and three ketamine medicine sessions (Tsang et al. Ther Adv Psychopharmacol 2023;13:1–14). Predictors of treatment responses within this model are unclear.

**Objectives:**

1. Evaluate pre–post changes in PHQ-9, GAD-7, PCL-5, and B-IPF;
2. Assess whether baseline severity predicts improvement;
3. Examine effects of concurrent pharmacotherapy or prior psychotherapy;
4. Test whether Adverse Childhood Experiences (ACEs) predict treatment outcomes.

**Methods:**

We conducted a retrospective cohort analysis of 305 participants enrolled in the 12-week RTT-KAT program (2018–2023), using validated scales (PHQ-9, GAD-7, PCL-5, BIPF) administered at baseline and post-program (Figure 1). Paired t-tests with 95% CI and Cohen’s *d.* Linear regressions assessed whether baseline severity predicted improvement, while ANCOVA of post-scores adjusted for baseline and pharmacotherapy/psychotherapy status. ACEs were evaluated using Pearson correlation and Welch’s t-tests (ACE ≥4 vs <4). Multiple analyses accounted for multiple comparisons with α=0.05.

**Results:**

Significant reductions were observed for PHQ-9, GAD-7, PCL-5 (all *p*<0.001, *d*>0.8) and moderate improvement for B-IPF (*d*=0.5; Figure 1). Higher baseline severity predicted greater absolute improvement across scales (negative β, *p*<0.001; Figure 2). After adjusting for baseline severity, concurrent pharmacotherapy and prior psychotherapy showed no effect on depression or anxiety. Pharmacotherapy predicted higher adjusted PTSD post-scores (β=4.48, 95% CI 0.72–8.24, *p*=0.020) and a non-significant trend toward poorer functioning (*p*=0.064). ACEs showed no significant correlation with treatment response (|r|<0.08, all *p*>0.20). Threshold comparisons (ACE ≥4 vs <4) also showed no group differences (all *p*>0.13). All confidence intervals included zero, indicating no evidence of either linear or threshold associations. Substance-use history was unrelated to outcomes (all p>0.20). Similarly, current alcohol, cannabis, or tobacco use did not significantly affect post-treatment outcomes once baseline severity was controlled.

**Image 1:**
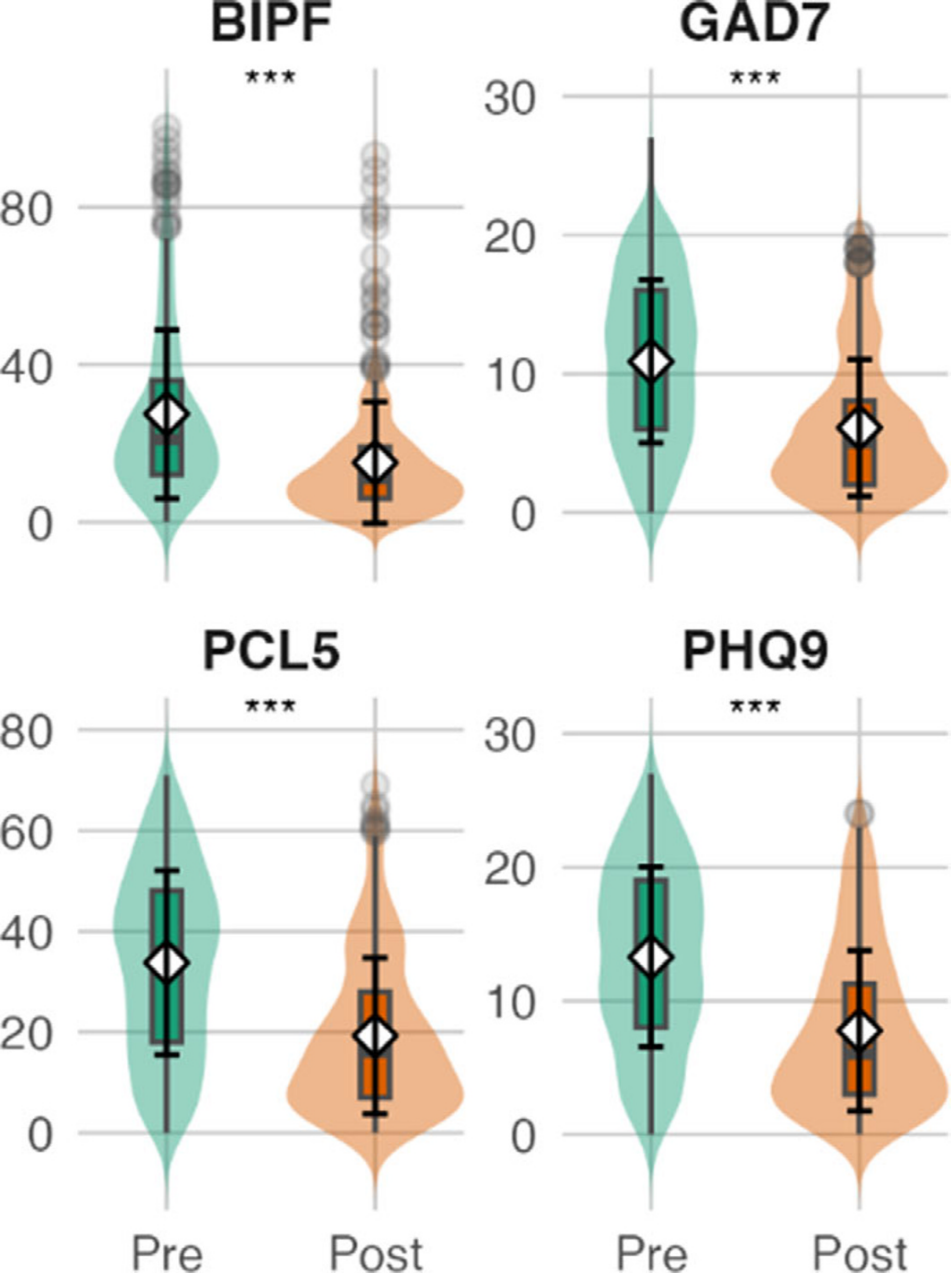
Image 1: Long description.

**Image 2:**
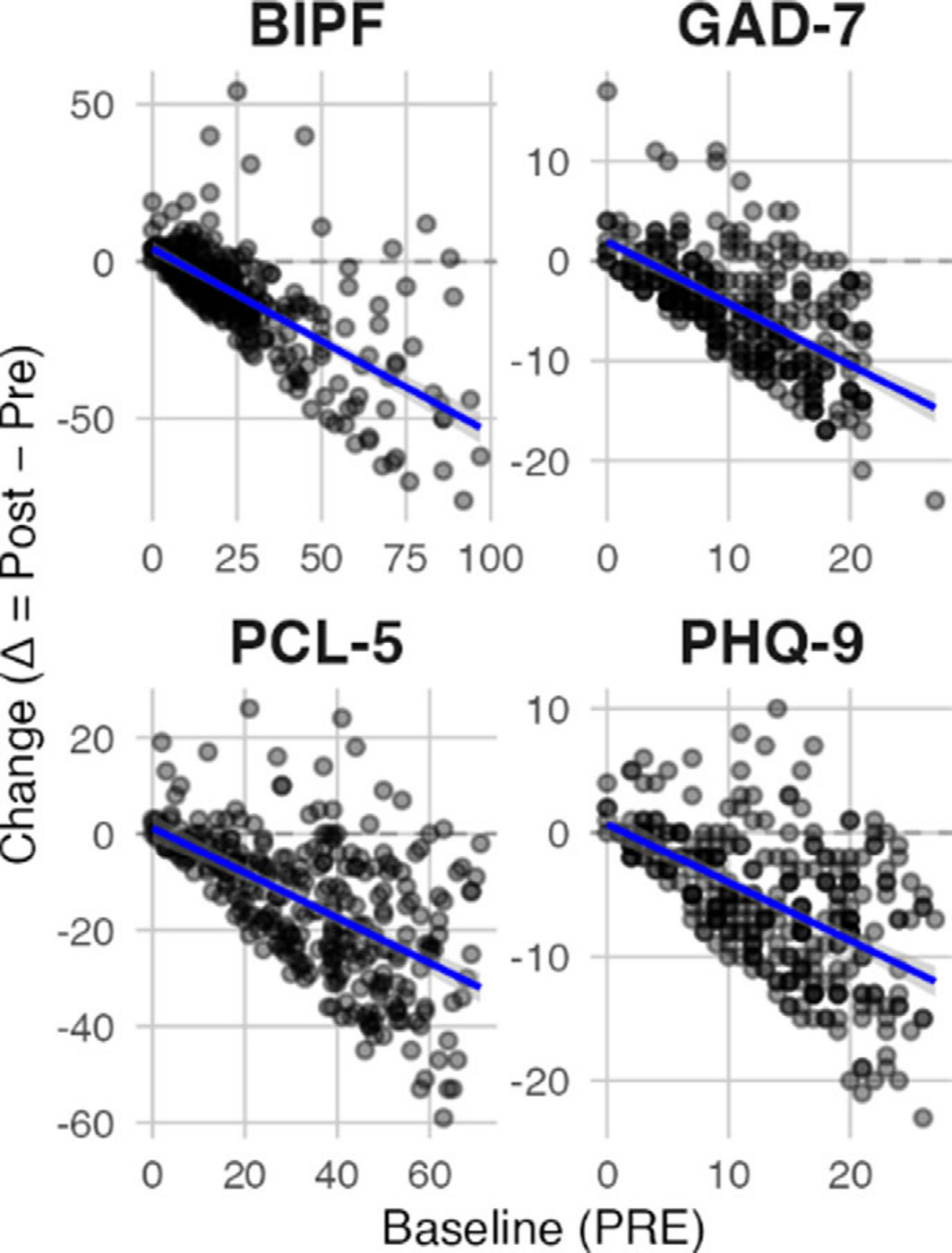
Image 2: Long description.

**Conclusions:**

RTT-KAT was associated with significant reductions in depression, anxiety, and PTSD. Improvement was greatest among those with higher baseline severity. Neither ACEs, prior psychotherapy, pharmacotherapy nor substance-use history predicted outcomes. Overall, baseline severity emerged as the most consistent predictor of clinical improvement, underscoring the potential of group-based KAT as a broadly effective and accessible intervention for individuals with high symptom burden.

**Disclosure of Interest:**

None Declared

## Long descriptions

### Image 1: Long description

The figure consists of four panels arranged in a two-by-two grid. Each panel contains two violin plots comparing Pre (teal) and Post (orange) measurements. All panels feature three asterisks at the top center, indicating statistical significance.

* Top-Left Panel (B I P F): The y-axis ranges from 0 to 80. The Pre distribution is wider around 30, with a white diamond mean marker at approximately 28. The Post distribution is narrower and lower, with a mean marker near 15. Both show several outlier circles extending toward the top of the axis.

* Top-Right Panel (G A D 7): The y-axis ranges from 0 to 30. The Pre distribution is elongated with a mean near 11. The Post distribution is wider at the base with a mean near 6. Outliers are present in the Post group.

* Bottom-Left Panel (P C L 5): The y-axis ranges from 0 to 80. The Pre distribution is centered around a mean of 35. The Post distribution shows a significant shift downward with a mean near 20 and a concentration of data at the lower end.

* Bottom-Right Panel (P H Q 9): The y-axis ranges from 0 to 30. The Pre distribution has a mean near 13. The Post distribution is wider at the bottom with a mean near 8.

In all panels, the internal box plots show a decrease in the median and interquartile range from the Pre to the Post condition.

Navigate back to Image 1:.

### Image 2: Long description

The x-axis is labeled Baseline (P R E) and the y-axis is labeled Change (delta equals Post minus Pre). All four panels feature a horizontal dashed line at zero on the y-axis, representing no change. Each panel contains a scatter plot of semi-transparent gray dots and a blue linear regression line with a light gray confidence interval band.

* Top-left panel B I P F: The x-axis ranges from 0 to 100 and the y-axis from -50 to 50. The blue line shows a strong linear decrease starting near (0, 5) and ending near (100, -50).

* Top-right panel G A D-7: The x-axis ranges from 0 to 20 and the y-axis from -20 to 10. The blue line shows a linear decrease starting near (0, 2) and ending near (25, -15).

* Bottom-left panel P C L-5: The x-axis ranges from 0 to 60 and the y-axis from -60 to 20. The blue line shows a linear decrease starting near (0, 0) and ending near (75, -30).

* Bottom-right panel P H Q-9: The x-axis ranges from 0 to 20 and the y-axis from -20 to 10. The blue line shows a linear decrease starting near (0, 1) and ending near (25, -12).

In all panels, higher baseline scores are associated with greater negative changes (reductions) in the measured metrics.

Navigate back to Image 2:.

